# Egg Consumption and Human Cardio-Metabolic Health in People with and without Diabetes

**DOI:** 10.3390/nu7095344

**Published:** 2015-09-03

**Authors:** Nicholas R. Fuller, Amanda Sainsbury, Ian D. Caterson, Tania P. Markovic

**Affiliations:** The Boden Institute of Obesity, Nutrition, Exercise & Eating Disorders, Charles Perkins Centre, The University of Sydney, NSW 2006, Australia; E-Mails: amanda.salis@sydney.edu.au (A.S.); ian.caterson@sydney.edu.au (I.D.C.); tania.markovic@bigpond.com (T.P.M.)

**Keywords:** dietary cholesterol, eggs, type 2 diabetes mellitus, cardiovascular disease

## Abstract

The guidelines for dietary cholesterol and/or egg intake for both the general population and those at higher risk of cardiovascular disease (for example, people with type 2 diabetes mellitus (T2DM)) differ between countries, and even for different specialist societies in a country. The disparity between these guidelines is at least in part related to the conflicting evidence as to the effects of eggs in the general population and in those with T2DM. This review addresses the effect of eggs on cardiovascular disease (CVD) risk from both epidemiological research and controlled prospective studies, in people with and without cardio-metabolic disease. It also examines the nutritional qualities of eggs and whether they may offer protection against chronic disease. The evidence suggests that a diet including more eggs than is recommended (at least in some countries) may be used safely as part of a healthy diet in both the general population and for those at high risk of cardiovascular disease, those with established coronary heart disease, and those with T2DM. In conclusion, an approach focused on a person’s entire dietary intake as opposed to specific foods or nutrients should be the heart of population nutrition guidelines.

## 1. Introduction

Despite our increased understanding of the pathophysiology of cardiovascular disease, there remains uncertainty regarding the role of dietary cholesterol and eggs in its pathophysiology. The guidelines for dietary cholesterol and/or egg intake for both the general population and those at higher risk of cardiovascular disease (for example, people with type 2 diabetes mellitus (T2DM)) differ between countries, and even for different specialist societies in a country. For example, the National Heart Foundation guidelines recommend that all Australians, including those with T2DM or metabolic syndrome, restrict their egg intake to six eggs or fewer per week [1]; the British Heart Foundation and Diabetes United Kingdom do not have a limit for dietary cholesterol or egg consumption [2]; and the American Diabetes Association (ADA) until very recently had a limit on total cholesterol consumption for both the general population and those with T2DM of 300 mg per day [3], with one egg containing approximately 200 mg cholesterol. This guideline has since been changed and there is no longer a limit on dietary cholesterol intake [4]. The American Heart Foundation and the American College of Cardiology have also abolished their dietary cholesterol restrictions, but another group in the United States, the National Lipid Association (NLA), has since revised their guidelines and is recommending <200 mg per day of dietary cholesterol for those with dyslipidaemia [5,6,7]. The disparity between these guidelines is at least in part related to the conflicting evidence as to the effects of eggs in the general population and in those with T2DM. This review addresses the effect of eggs on cardiovascular disease (CVD) risk from both epidemiological research and controlled prospective studies, in people with and without cardio-metabolic disease. Cardio-metabolic risk refers to risk factors associated with increased risk of cardiovascular disease and metabolic disease, and these two conditions are in turn related. Metabolic disease or disorders include T2DM, insulin resistance, hypertension and dyslipidaemia. The review also examines the nutritional qualities of eggs and whether they may offer protection against chronic disease.

## 2. Epidemiological Evidence Suggesting That a High-Egg Diet is Safe for the General Population but has Adverse Cardio-Metabolic Effects, Particularly in those with Diabetes Mellitus

Epidemiological studies to date have indicated very little association between a high egg intake and cardiovascular disease or mortality in the general population; however, evidence suggests an adverse effect in sub-groups of the population, notably in those with diabetes mellitus. A summary of each of the epidemiological studies is provided in Table 1. Some of these studies of longer-term follow up or larger sample size will be reviewed below in greater detail.

**Table 1:** Summary of epidemiological evidence regarding egg consumption, cardiovascular disease, and incidence of diabetes.

Study	Design	Association between Egg Consumption and Cardiovascular Disease	Association between Egg Consumption and Incidence of Diabetes
Framingham Study and Offspring study [8,9]	24 year follow up of 912 and a prospective cohort of 2879 American participants	No association between egg intake and subsequent development of CHD	Intake of eggs associated with incidence of type 2 diabetes with some dietary pattern scores
Italian case-control study [10]	287 cases with AMI and 649 controls, Italian women, conducted over 5 years	No association between egg consumption of greater than 2 eggs/week and nonfatal myocardial infarction	Not reported
Finnish Study [11]	14 year follow up of 5133 Finnish men and women aged 30–69 years	No difference in egg consumption between individuals who developed fatal coronary heart disease and those who did not	Not reported
Oxford Vegetarian Study [12]	14 year follow up of 11,140 English vegetarians and meat eating participants	>6 eggs/week associated with increased mortality from ischemic heart disease	Not reported
Adventist Health Study [13]	6 year follow up of 34,192 vegetarian and non-vegetarian American Seventh Day Adventists	Consuming >2 eggs/week presents no difference in risk of developing CHD compared to consuming <1 eggs/week	Not reported
Nurses Health [14]	14 year follow up of 80,082 American women aged 39–54 years	No association between consumption of up to 1 egg/day and risk of CHD or stroke; higher egg consumption was associated with increased CHD risk in people with diabetes	Not reported
Health Professionals Follow-up [14]	14 year follow up of 37,851 American men aged 40–75 years	No association between consumption of up to 1 egg/day and risk of CHD or stroke; higher egg consumption was associated with increased CHD risk in people with diabetes	Not reported
Japanese case-control study [15]	660 cases with AMI and 1277 controls, Japanese men and women aged 40–79 years	No association between egg intakes up to 4 or more/week and incidence of AMI	Not reported
NIPPON DATA80 [16]	14 year follow up of 5186 women and 4077 men, all Japanese aged 30 years and over	No effect of egg consumption on risk of fatal CHD events, stroke and cancer in men or women consuming ≥2 eggs/day; increased risk of all cause mortality in women for ≥1 egg/day	Not reported
Japan Public Health Centre-based study [17]	21 year follow up of 90,735 Japanese male and female participants aged 40–69 years	Total cholesterol levels were significantly related to an increased risk of CHD, however consumption of eggs almost daily was not associated with CHD risk in middle-aged Japanese men and women	Not reported
Greek EPIC diabetic subgroup [18]	11 year follow up of 1013 Greek adults with diabetes	Positive association with increased egg consumption and cardiovascular mortality in people with diabetes	Not reported
NHANES I [19]	20 year follow up of 9734 American adults aged 25 to 74 years	No significant difference between consuming >6 eggs/week compared to <1 egg/week in any stroke, ischemic stroke or coronary artery disease; consumption of >6 eggs/week was associated with an increased risk of CHD in people with diabetes	Not reported
Physician’s Health [20,21,22]	20 year follow up of 21,327 American male participants aged 40 years and over and 36,295 American women aged 45 years	Egg consumption did not increase CVD risk, but consumption of ≥7/week was associated with a 23% increased risk of all cause mortality and in a separate study of the same cohort, a 28% increase risk of heart failure; consumption of ≤6 eggs/week did not increase the risk of death from all causes	Men who ate 5–6 eggs/week had a 46% higher risk of developing type 2 diabetes than no eggs, and 58% higher for ≥7 eggs/week; women who ate 2–4 eggs a week had a 19% higher risk, and 77% higher for ≥7 eggs/week. In both groups, there was a significantly increased risk of developing type 2 diabetes with increasing egg consumption
INTERHEART (A Global Case-Control Study of Risk Factors for Acute Myocardial Infarction) [23]	Global study reporting on 5761 patients who have had a heart attack and 10,646 controls free of heart disease, recruited over 4 years	Western dietary pattern (characterised by higher intakes of fried foods, salty snacks and meat) was shown to be associated with an increased risk of heart attack; no association between eggs and heart attack risk	Not reported
Atherosclerosis Risk in Communities (ARIC) [24]	11 year follow up of 15,792 African American and white American men and women aged 45–64 years	23% increased risk of heart failure for each extra serving of eggs/day, up to 7 eggs/week	Not reported
Insulin Resistance Atherosclerosis Study [25]	Prospective cohort of 880 American individuals with normal glucose tolerance or impaired glucose tolerance	Not reported	High intake of a dietary pattern that included eggs (as well as red meat, low fibre bread and cereal, dried beans, fried potatoes, tomato, vegetables, cheese and cottage cheese, and low in wine) was associated with developing type 2 diabetes
Cardiovascular health study [26]	Prospective study of 3898 American older adults (>65 years) followed for an average of 11.3 years	Not reported	There was no association between egg consumption or dietary cholesterol intake and risk of developing type 2 diabetes
Health ABC Study [27]	9 year follow up of 1941 70–79 years old Americans	Dietary cholesterol and consumption of ≥3 eggs/week was associated with increased CVD risk only in older adults with type 2 diabetes (but not in those without type 2 diabetes)	Not reported
NHANES III [28]	9 year follow up of 20,050 American adults (17 years and over)	No association between egg intake (>7/week compared to <1/week) and CHD mortality	Not reported
Chinese cohort study [29]	Data from 2849 Chinese adults (20 years and over)	Not reported	Egg consumption was significantly and positively associated with diabetes risk. The OR of diabetes associated with egg consumption <2/week, 2–6/week, and ≥1/day in the total sample were 1.00, 1.75, 2.28 respectively. These associations were stronger in women compared to men
The SUN Project [30]	6 year follow up of 14,185 Mediterranean university students	No association between egg consumption and the incidence of CVD for the highest (>4 eggs/week) *versus* the lowest (<1 egg/week) category of egg consumption	Not reported
Case-control study [31]	234 Lithuanians aged 35–86 years with a newly confirmed diagnosis of type 2 diabetes according to WHO criteria, and 468 controls	Not reported	Participants who consumed >5 eggs/week had a higher risk (threefold) of type 2 diabetes than those who consumed <1 egg/week
Malmo Diet and Cancer Cohort [32]	Prospective cohort including 27,140 Swedish participants (45–74 years) during a 12 year follow up	Not reported	Highest quintiles of egg intake associated with increased risk of developing type 2 diabetes
Mediterranean cohort—the SUN project [33]	Prospective cohort of 15,956 participants from Spanish population (average age 38.5 years) during 6.6 years (median) follow up	Not reported	Egg consumption was not associated with the development of diabetes, comparing the highest (>4 eggs/week) with the lowest (<1 egg/week) quartile of egg consumption
The Northern Manhattan Study [34]	1429 American adults with carotid ultrasounds followed for 11 years	Egg consumption was inversely associated with carotid intima media thickness. For every additional egg consumed/week, risk of plaque decreased by 11%	Not reported
The Kuopio Ischaemic Heart Disease Risk Factor Study [35]	2332 men from Finnish population (42–60 years) during 19.3 years follow-up	Not reported	Higher egg intake was associated with a 38% lower risk of developing type 2 diabetes compared to those in the lowest group of egg intake
Meta-Analysis/Systematic Review regarding egg consumption, cardiovascular disease, and incidence of diabetes			
Cardiovascular diseases and diabetes meta-analysis [36]	14 studies (320,778 participants):11 prospective, 1 case-control and 2 cross-sectional studies. Sample size ranged from 488 to 117,943. Follow-up time from 6.1 to 20 years	Positive dose-response association between egg consumption and risk of CVD. A 19% increased risk of CVD in highest egg consumption compared to lowest egg intake. A sub group (participants with diabetes) found to have a further increased risk of CVD (RR 1.83)	There was a dose-response positive association between egg consumption and risk of diabetes
Dose-response meta-analysis of prospective cohort studies [37]	8 articles with 17 reports (9 for CHD and 8 for stroke)	No significant association found between egg consumption up to 1 egg/day and risk of CHD or stroke. In a subgroup analysis of people with diabetes, higher egg consumption (up to 1 egg/day) associated with a higher risk of CHD but lower risk of haemorrhagic stroke	Not reported
Cardiovascular disease and diabetes systematic review and meta-analysis [38]	22 independent cohorts from 16 studies. Number of participants ranged from 1600 to 90,735. Follow-up time ranged from 5.8 to 20 years	Consuming ≥1 egg/day was not associated with risk of overall CVD, ischemic heart disease, stroke or mortality. In a subgroup population (people with diabetes), those who ate eggs >once a day were 1.69 times more likely to develop CVD co-morbidity	Those who ate ≥1 egg/day (compared to those who never ate eggs) were 42% more likely to develop type 2 diabetes
Tran *et al.* systematic review [39]	8 epidemiological studies that examined the risk of developing type 2 diabetes mellitus. 6 of the studies evaluated egg consumption, whilst 2 of the studies evaluated dietary patterns that included eggs	Not reported	4 of the 8 studies found a significant association between diabetes risk and egg consumption.

Abbreviations: ABC, ageing and body composition; AMI, acute myocardial infarction; ARIC, atherosclerosis risk in communities; CHD, coronary heart disease; CVD, cardiovascular disease; EPIC, European prospective investigation into cancer and nutrition; NHANES, national health and nutrition examination survey; OR, odds ratio; RR, relative risk.

Earlier evidence as to the effect of egg intake on cardio-metabolic outcomes comes from the Framingham study (follow up of 24 years), which aimed to determine factors related to the development of cardiovascular disease. In doing so it addressed the effect of dietary intake (including egg consumption) on circulating cholesterol levels and on the incidence of coronary heart disease in a free living population in Framingham, MA, USA [8]. Egg intake in this population ranged from 0 to 24 eggs per week in males and from 0 to 19 per week in females, with an average egg consumption of 5.9 per week for males and 3.8 per week for females. Results showed no significant association between the number of eggs consumed with all-cause mortality, total coronary heart disease, myocardial infarction, or angina pectoris. Furthermore, a low *versus* high egg consumption had no effect on blood cholesterol level. This finding supported the data from intervention studies conducted at the same time (late 1970s) showing no effect of egg feeding in the general population [40,41,42]. Importantly, the Framingham study also suggested that focus should be placed on a person’s entire dietary intake rather than egg or cholesterol intake alone, because circulating cholesterol distribution curves of the subjects according to tertiles of egg or cholesterol intake were more or less identical. This study has been supported by several other large epidemiological studies conducted later. Hu and colleagues reported on egg consumption and risk of cardiovascular disease in two large prospective cohort studies examining both males from the Health Professionals Follow-up Study (*n* = 37,851) and females from the Nurses’ Health Study (*n* = 80,082) [43]. Both studies found a decline in the average egg consumption from 2.3 per week in 1986 to 1.6 eggs per week in 1990 for males, and a decline from 2.8 eggs per week in 1980 to 1.4 eggs per week in 1990 for females. This coincided with the increased emphasis in the USA during that same period [44,45], to restrict dietary cholesterol intake to less than 300 mg per day and limit egg consumption due to the high dietary cholesterol content of eggs. Egg consumption (one egg per day) had no significant association with nonfatal myocardial infarction or mortality from coronary heart disease, or risk of total stroke or its subtypes, whether or not subjects with diabetes or hypercholesterolemia were included in the analyses [43]. However, when examining subgroups of the population, a positive association between a higher egg intake and relative risk of coronary heart disease was found for those with diabetes [43]. Results were similar in the Physicians Health Study (*n* = 21,327), in that egg consumption (<7/week) was not associated with myocardial infarction, stroke or total mortality in male physicians [21]. However, consumption of greater than or equal to seven eggs per week was associated with a greater risk of mortality for this entire cohort of male physicians. This positive correlation to mortality with a higher intake of eggs (≥7 eggs/week) was evident more so in those with diabetes [21]. At the time when the Physicians Health Study was conducted (as well as the majority of the other epidemiological studies), a public health campaign (which was emphasised in the early 1980s) was advising people to limit their cholesterol intake (including their consumption of eggs) [44,45]. Individuals (particularly physicians) consuming a high number of eggs during that time may have been less likely to have been following healthy dietary and lifestyle advice in general. Indeed, in this study [21], male physicians consuming a higher intake of eggs were also following other unhealthy behaviours including reduced frequency of exercise and increased smoking, and had a higher prevalence of diabetes and hypertension [21].

Epidemiological data from studies conducted in Japan [15,16,17], Italy [10] and Finland [11] and systematic and meta-analytic reviews [37,38] also support the above-mentioned data from the United States (Framingham Study [8], Health Professionals Follow-up Study, Nurses’ Health Study [43]) as well as data from other United States studies listed in Table 1 [13,19,28], in that egg consumption showed no significant association with the risk of coronary heart disease or cardiovascular heart disease in the general population. However, again, as seen with some studies conducted in the United States [21,27,43], this result was not consistent when analysing sub-groups of the population, such as in those with self-reported diabetes [17,18]. With respect to egg consumption and incidence of diabetes, again there are inconsistencies. Despite most studies suggesting an increased association of diabetes incidence and egg consumption [20,29,31,32,36,38], some studies show no association between egg consumption and risk of developing T2DM [26,33], with one study even showing a higher egg intake being associated with a 38% lower risk of developing T2DM [35].

While the above-mentioned studies show no overall effect of eggs on CVD (at least up to an intake of six eggs per week), and an increased incidence of T2DM with increased egg consumption, when considering stroke, some of these epidemiological studies have shown a significant inverse relationship between a high egg consumption and reduced risk of total and haemorrhagic stroke, and stroke mortality [19,28,46]. This data provides circumstantial and weak evidence that eggs may have protective effects against certain pathologies. 

In summary, there are inconsistencies in the findings between these prospective cohort studies in terms of the risk of CVD and mortality, and incidence of diabetes mellitus. Most studies show no association between egg consumption and CVD risk in a healthy population [8,10,11,13,17,19,28,30,34,47], while others suggest an increased risk of CVD with higher egg consumption (≥7 eggs per week) [12,16,21,24]. With respect to the incidence of diabetes with egg consumption, most studies suggest an increased association of diabetes incidence and egg consumption [20,29,31,32,36,38], some studies show no association between egg consumption and risk of developing T2DM [26,33], and one study shows a protective effect of higher egg intake and incidence of T2DM [35]. Conversely, the risk of stroke appears to be lower with higher egg consumption [19,28,46]. Similar discrepant findings are seen in subgroups of the population and specifically in people with diabetes mellitus, with some studies showing no increased risk in CVD with egg consumption [8,17,28,35], but the majority suggesting that a higher egg intake (usually ≥7 eggs per week) may increase the risk of CVD in this group [18,19,21,27,43]. An important limitation of these epidemiologic studies in general is the presence of confounding factors that have a known effect on coronary artery disease and cardiovascular heart disease that may not have been accounted for. Despite adjusting for some confounding factors in statistical models in the Physicians Health Study, detailed dietary data (total energy intake and saturated fat) and other important variables (markers of insulin resistance and lipids) that predict the onset of cardiovascular disease were not taken into account [21]. Intake of energy, total fat, fruit or wholegrains, as well as body mass index and family history, were only controlled for in a minority of the above-mentioned epidemiological studies [28,43]. These limitations highlight the need for controlled, prospective studies to determine the impact of eggs *per se* on cardio-metabolic health. Importantly, it is now known that dietary cholesterol is not the principal factor affecting circulating cholesterol levels, with the main determining dietary factors being saturated and trans-fat intake [48,49], for which only one [28] of these epidemiological studies adjusted. In this study that adjusted for saturated fat intake [28], there was no increased risk of coronary heart disease mortality or stroke in those eating greater than six eggs per week compared to those eating one to six eggs per week.

## 3. Controlled Prospective Studies of the Effects of a High Egg Diet on Dietary Cholesterol Intake and Circulating Cholesterol Levels

### 3.1. The Relationship between Dietary and Circulating Cholesterol

The effect of dietary cholesterol intake on circulating cholesterol level is small. A meta-analysis of cholesterol feeding studies including both healthy populations and populations with cardio-metabolic disease, using a variety of sources of dietary cholesterol (including eggs) showed that for every 100 mg per day increase in dietary cholesterol intake, circulating total cholesterol increased by 0.06 mmol/L, high-density lipoprotein (HDL) increased by 0.008 mmol/L, and the ratio of total to HDL cholesterol increased by 0.020 [50]. One large egg contains approximately 200 mg of dietary cholesterol, so consuming an egg a day would be expected to increase total circulating cholesterol levels by approximately 0.12 mmol/L [50]. While mean changes in lipoproteins in response to dietary cholesterol are small, considerable heterogeneity has been observed in circulating cholesterol responses to dietary cholesterol [51]. For example, there appears to be less efficient absorption of dietary cholesterol in those who have obesity and insulin resistance, when compared to those who are lean and insulin sensitive [52,53]. However, meta-analyses comparing the effects of dietary cholesterol and fat on circulating lipid and lipoprotein levels reveal that dietary saturated and trans-fat elicit much stronger effects, and taking into consideration their higher percentage energy contribution in the diet relative to dietary cholesterol, saturated and trans-fat are the major contributors to circulating total and low-density lipoprotein (LDL) cholesterol levels [48,49,54]. For every 2.8-gram per day reduction in saturated fat intake, total cholesterol reduces by approximately 0.08 mmol/L. Therefore, while increasing egg intake by one egg per day would be expected to increase total cholesterol by approximately 0.12 mmol/L, a concomitant reduction in saturated fat intake by 6 g per day (the amount of saturated fat in a tablespoon of butter, for example) would be expected to reduce circulating cholesterol levels by a similar amount.

### 3.2. Studies Conducted in the General Population

Prospective controlled studies conducted in the general population (that is, in those that are relatively healthy without cardio-metabolic disorders) have shown differing effects of egg consumption on CVD risk. There have been numerous cholesterol feeding studies conducted in a free-living general population over the last 50 years and some of these studies are referenced in the following section. However, a summary of only those controlled prospective studies conducted in the general population since the meta-analytic review performed by Weggemans and colleagues [50] is provided in Table 2.

In some studies in which additional cholesterol (in the form of eggs) has been added to peoples’ diet under strict control, there have been increases in circulating total and LDL cholesterol noted [55,56,57], whilst in other such studies there have been no changes [40,41,42,58,59,60]. In some studies, circulating HDL cholesterol levels significantly increased with the addition of eggs to the diet [61,62,63], which was also found in the meta-analytic review of dietary cholesterol feeding in 556 subjects from 17 heterogeneous studies using both eggs and high cholesterol products [50]. However, in that review the authors reported the adverse coronary risk finding of an increase in the ratio of total to HDL cholesterol by 0.02 units [50]. The majority of these studies (15 of 17 of them) involved subjects from an otherwise healthy population without metabolic disorders, but one study included those with type 1 diabetes mellitus (T1DM) and another included subjects with hypercholesterolemia and hyperlipidaemia. While there was a small but statistically significant adverse change in the total to HDL cholesterol ratio overall, five of the 17 studies showed no adverse effects of cholesterol feeding on the lipid profile, six studies showed equivocal effects, and only six studies showed adverse effects. More importantly, this change in lipid profile appeared to be dependent on the quality of the diet prescribed, or background diet of the population group [50,64,65]. This meta-analytic review [50] found that in subjects who were fed a high cholesterol diet and who had a background diet that was low in saturated fats (a polyunsaturated to saturated fat ratio > 0.7), the increase in LDL cholesterol was less apparent than in those studies in subjects in whom the background diet was high in saturated fats (a polyunsaturated to saturated fat ratio ≤ 0.7) [50]. Thus, these observations suggest that a person consuming a higher dietary cholesterol diet in the context of a diet lower in saturated fat is unlikely to experience any adverse effect on circulating lipids.

**Table 2:** Effect of egg consumption on cholesterol levels in the general population.

Reference	Study Details	Cholesterol/Egg Intake	Effect on Lipids from High Cholesterol or Egg Intake
Greene *et al*. 2005 [66]	42 healthy postmenopausal women and men > 60 years	3 eggs/day for 1 month (compared to egg substitute)	LDL and HDL cholesterol levels increased; LDL:HDL and TC:HDL ratios did not change
Katz *et al*. 2005 [67]	49 healthy adults	2 eggs/day for 6 weeks (compared to oats breakfast)	No effect on total cholesterol or endothelial function
Goodrow *et al*. 2006 [68]	33 adults > 60 years	1 egg/day for 5 weeks (compared to egg substitute)	No increase in total cholesterol, LDL cholesterol or HDL cholesterol levels
Harman *et al*. 2008 [69]	45 overweight or obese adults (18–55 years)	2 eggs/day as part of an energy restricted weight loss diet (compared to no eggs/day)	Decreased total and LDL cholesterol
Mutungi *et al*. 2008 [61]	28 overweight or obese men (40–70 years)	3 eggs/week as part of a CHO restricted weight loss diet (compared to egg substitute)	Increased HDL cholesterol; no change in LDL cholesterol levels
Rueda *et al*. 2013 [70]	73 university students	Breakfast with eggs 5 times/week for 14 weeks (compared to breakfast without eggs) Egg breakfast contained 400mg more cholesterol than the breakfast without eggs	No significant differences in total, LDL cholesterol or HDL cholesterol between the two groups
Clayton *et al*. 2015 [71]	25 healthy young adults (18–35 years)	2 eggs per day (compared to a bagel breakfast) for 12 weeks	No impact on total cholesterol, HDL cholesterol or LDL cholesterol levels The egg breakfast led to improvements in triglyceride levels

Abbreviations: CHO, carbohydrate; HDL, high density lipoprotein; LDL, low density lipoprotein; TC, total cholesterol.

Of the more recent studies completed (Table 2), five of the seven studies have shown no adverse effects on the lipid profile following a high egg intake [66,67,68,70,71] and two have shown improvements in circulating lipids with an increased egg consumption [61,69].

### 3.3. Studies Conducted in people with High Risk of Cardiovascular Disease, Established Coronary Heart Disease, or with Diabetes Mellitus

In contrast to the variable effects of cholesterol feeding on circulating lipid profiles in the general population (that is, in those that are otherwise healthy and without cardio-metabolic disorders), with some studies showing an increase in the ratio of total to HDL cholesterol and LDL cholesterol, but others showing no adverse effects, in people with a high risk of cardio-metabolic disease the effects of a high egg diet appear generally positive. As there has been only a small number of well-designed studies conducted in such a population (that is, in people with high risk of cardiovascular disease or T2DM, established coronary heart disease, or with diabetes mellitus), these will be reviewed in greater detail. Six of these studies have been conducted in individuals at high risk of cardiovascular disease or T2DM [52,53,72,73,74,75,76,77,78], one in those with established coronary heart disease [79], and three in those with T2DM [80,81,82]. Of these, three studies have shown beneficial effects of a high egg diet on cardio-metabolic risk factors with respect to the comparator or control group [72,73,74,75,81,82], five have shown no adverse effect [53,76,78,79,80], and two have shown a detrimental effect, but only in sub-groups of the population being investigated [52,77]. A summary of each of the controlled prospective studies conducted in people with cardio-metabolic disease is provided in Table 3.

**Table 3:** Effect of egg consumption on cholesterol levels in those with cardio-metabolic disease, including type 2 diabetes mellitus.

Reference	Study Details	Cholesterol/Egg Intake	Effect on Lipids from Increased Cholesterol or Egg Intake
Edington *et al.* 1987 [76]	168 adults with hyperlipidaemia	2 eggs/week or 7 eggs/week as part of a low fat, high fibre diet for 8 weeks	No change to total, LDL or HDL cholesterol
Knopp *et al.* 1997 [77]	161 adults with hypercholesterolemia or hyperlipidaemia	2 eggs/day as part of an American Heart Association diet for 6 weeks	Increased LDL and HDL cholesterol levels; Adults with only high cholesterol had only non-significant increases in LDL cholesterol
Knopp *et al.* 2003 [52]	197 adults with insulin resistance	4 eggs/day for 4 weeks	Increased LDL cholesterol; This increase in LDL cholesterol was less in insulin resistant individuals compared to insulin sensitive individuals
Tannock *et al.* 2005 [53]	201 lean insulin-sensitive adults and lean or obese insulin resistant adults	4 eggs/day as part of a low fat diet for 4 weeks	HDL cholesterol increased in all subjects; non HDL cholesterol levels increased in lean insulin-sensitive subjects but not insulin-resistant subjects
Njike *et al.* 2010 [78]	40 adults with hyperlipidemia	3 and then 2 eggs/day (acute and sustained phase) for 6 weeks (compared to sausage/cheese breakfast sandwich and egg substitute)	No change to total, LDL or HDL cholesterol. No detrimental effects on flow mediated dilatation
Pearce *et al.* 2011 [81]	65 adults with type 2 diabetes or impaired glucose tolerance	2 eggs/day as part of an energy restricted, high protein diet for 12 weeks (compared to animal protein substitute)	Increased HDL cholesterol levels
Blesso *et al.* 2013 [73,74,75] and Andersen *et al.* 2013 [72]	37 adults with metabolic syndrome	3 eggs/day as part of carbohydrate restricted diet for 12 weeks (compared to egg substitute)	No change in total or LDL cholesterol; Increased HDL cholesterol; Increased LDL particle size
Fuller *et al.* 2015 [80]	140 overweight or obese adults with impaired glucose tolerance or type 2 diabetes	2 eggs/day for 6 days/week as part of a 3 month weight maintenance diet (compared to <2 eggs/week)	No difference in change in HDL cholesterol, total cholesterol or LDL cholesterol levels between the two groups
Katz *et al.* 2015 [79]	32 adults with CAD	2 eggs/day for 6 weeks	Daily consumption of eggs showed no adverse effects on total cholesterol levels compared to a high-carbohydrate breakfast

Abbreviations: CAD, coronary artery disease; HDL, high density lipoprotein; LDL, low density lipoprotein.

In a study investigating the effect of high egg intake (three eggs per day) *versus* egg substitute (which is comprised of 99% egg white and contains no cholesterol or fat) in those with metabolic syndrome, improvements in dyslipidemia were noted for both groups when accompanied by a three-month weight reduction program. However, reductions in circulating concentrations of the inflammatory markers tumour necrosis factor alpha (TNF-α) and serum amyloid A (a protein secreted in response to inflammatory stimuli) only occurred in the egg group [74]. Thus the high egg diet had a beneficial effect in reducing inflammation in this population with metabolic syndrome.

One study has been conducted in people with established coronary heart disease, and in contrast to the majority of studies, the primary outcome was endothelial function, assessed by flow-mediated dilatation. The authors found no difference in flow-mediated dilatation or circulating lipid levels between subjects that were following a high egg diet of two eggs per day compared to those following a high carbohydrate breakfast or a breakfast containing egg substitute [79]. One other study in which subjects with hyperlipidaemia were prescribed three eggs during the acute phase and two hard-boiled eggs during the sustained phase for breakfast along with their habitual diet, found no detrimental effects on flow mediated dilatation or lipid profile when compared to baseline levels [78].

In an earlier study in those with either hypercholesterolemia or hyperlipidaemia, subjects followed the National Cholesterol Education Program (NCEP) Step I Diet for six weeks before being randomised to 2 eggs or egg substitute daily [77]. There was no difference between the hypercholesterolemia or hyperlipidaemia egg fed groups for change in LDL cholesterol, when compared to a control group not fed eggs. However, the authors also reported on within group changes and found that there were significant increases in LDL cholesterol relative to baseline in the hyperlipidaemic egg fed group, and significant increases in HDL cholesterol in both the hypercholesterolemia and hyperlipidaemic egg fed groups from baseline to 12 weeks [77]. However, an important limitation of this study is that the group on the high egg diet also had a significantly higher intake of saturated fat compared to the control group not fed eggs [77].

There have been only three controlled, prospective studies investigating the effects of a high egg diet specifically in people with T2DM, and only one study in people with T1DM [83]. This short-term study over three weeks examined cholesterol feeding in both subjects with and without T1DM. There was an increase in the ratio of LDL to HDL cholesterol over a three-week period for those with T1DM only when 800 mg of cholesterol was added to their diet daily (as a liquid supplement containing egg yolk) [83]. One of the studies conducted in subjects with T2DM was accompanied by a weight loss prescription, which may have counteracted any potential detrimental effects of eggs on cardiovascular markers. In that study, there was no difference in LDL cholesterol between the high (two eggs per day) and no egg diet groups, and those on a high egg diet had a significant increase in HDL cholesterol compared to the no egg diet [81]. The other two studies were conducted under weight maintenance conditions. Over the course of a three-month weight maintenance study examining the effects of a high (12 eggs per week) *versus* low-egg (<2 eggs per week) diet [80] in those with impaired glucose tolerance or T2DM, the findings were similar to those reported by Pearce *et al.* in their weight loss study [81]. During this study subjects were required to maintain their weight and activity level, with an emphasis placed on replacing saturated fat with poly- and mono-unsaturated fatty acids in the diet. No adverse changes in circulating lipid profiles were evident when compared to those following a low egg diet [80]. Lastly, in a study comparing the consumption of one egg per day for breakfast *versus* an oatmeal-based breakfast in those with T2DM, there was no difference in fasting plasma glucose between groups after a five-week period. Similarly to the study in subjects with metabolic syndrome [74], there was a significant reduction in the inflammatory marker TNF-α in the one egg per day group [82].

Thus, apart from one small study of short duration (three weeks) which showed an increase in the ratio of LDL to HDL cholesterol with the addition of 800 mg dietary cholesterol daily in people with T1DM, all other studies conducted to date in subjects with cardio-metabolic disease or T2DM, have shown either a positive or no adverse effect on cardiovascular risk factors from a high egg diet.

## 4. Positive and Negative Nutritional Qualities of Eggs

Eggs are very high in dietary cholesterol, and despite an increase in circulating LDL cholesterol levels seen in some but not all dietary cholesterol feeding studies, eggs do possess nutritional benefits that may have benefits on health outcomes and CVD risk.

Eggs contain carotenoids (lutein and zeaxanthin) recognised for their role in protecting against age-related macular degeneration and cataracts, as well as for their antioxidant and anti-inflammatory properties [75,84]. They provide arginine (a precursor to nitric oxide), which in turn causes blood vessels to dilate, thereby playing a key role in endothelial function [85], and folate, which may reduce the risk of T2DM and cardiovascular disease [86,87], and risk of neural tube defects during pregnancy [88].

Omega-3 fortified eggs may also serve a role in the diet, particularly for people with hypertriglyceridemia and those who avoid fish. Two studies have shown consumption of omega-3 supplemented eggs to be associated with a significant decrease in circulating triglycerides [89,90], consistent with the improvements in triglyceride levels seen with fish or fish oil consumption [91,92].

Eggs are a substantial source of choline, which is a known neurotransmitter involved in cognitive function [93], but dietary phosphatidylcholine is associated with the production of a proatherosclerotic metabolite, trimethylamine-*N*-oxide (TMAO) in a gut-flora dependent manner, and this has been associated with an increased risk of cardiovascular events [94]

However, to date the effect of long term egg intake on TMAO levels has not been assessed. Thus despite the potential for an adverse effect of the cholesterol in eggs on LDL cholesterol, it is conceivable that specific components of eggs could also contribute to favourable health outcomes and reduced CVD risk in people who consume a high egg diet. When eggs are included in the context of a healthy diet, these nutritional benefits could conceivably outweigh any adverse effects of eggs, albeit further well-controlled studies are required to test this.

## 5. Conclusions

Despite conflicting guidelines between countries regarding dietary cholesterol and specifically egg intake, the evidence suggests that a diet including more eggs than is recommended (at least in some countries) may be used safely as part a healthy diet in both the general population and for those at high risk of cardiovascular disease, those with established coronary heart disease, and those with type 2 diabetes mellitus. The background or intervention diet appears to be a key nutritional component. A high egg diet in the context of a background diet that is low in saturated fats (a polyunsaturated to saturated fat ratio > 0.7), or a diet that replaces saturated fats with poly- and mono-unsaturated fats, is likely to result in positive or no adverse changes in LDL cholesterol, and could be safely advised. Hence, an approach focused on a person’s entire dietary intake as opposed to specific foods or nutrients should be the heart of population nutrition guidelines.
